# Salience effects in information acquisition: No evidence for a top-down coherence influence

**DOI:** 10.3758/s13421-021-01188-9

**Published:** 2021-06-16

**Authors:** Arndt Bröder, Sophie Scharf, Marc Jekel, Andreas Glöckner, Nicole Franke

**Affiliations:** 1 School of Social Sciences, University of Mannheim, 68131 Mannheim, Germany; 2 Department of Psychology, University of Cologne, Cologne, Germany; 3 Department of Psychology, University of Hagen, Hagen, Germany

**Keywords:** Decision making, Information search, Coherence

## Abstract

The Integrated Coherence-Based Decision and Search (iCodes) model proposed by Jekel et al. (*Psychological Review,* 125 (5), 744–768, 2018) formalizes both decision making and pre-decisional information search as coherence-maximization processes in an interactive network. Next to bottom-up attribute influences, the coherence of option information exerts a top-down influence on the search processes in this model, predicting the tendency to continue information search with the currently most attractive option. This hallmark “attraction search effect” (ASE) has been demonstrated in several studies. In three experiments with 250 participants altogether, a more subtle prediction of an extended version of iCodes including exogenous influence factors was tested: The salience of information is assumed to have both a direct (bottom-up) and an indirect (top-down) effect on search, the latter driven by the match between information valence and option attractiveness. The results of the experiments largely agree in (1) showing a strong ASE, (2) demonstrating a bottom-up salience effect on search, but (3) suggesting the absence of the hypothesized indirect top-down salience effect. Hence, only two of three model predictions were confirmed. Implications for various implementations of exogenous factors in the iCodes model are discussed.

## Introduction

Decision making typically involves a pre-decisional process in which people actively search for potentially useful information about choice options. In laboratory tasks, computerized information-board designs have been used to monitor these search processes, and they are often seen as indicative of strategies used for information integration (Schulte-Mecklenbeck et al., 2019). For example, it is typically assumed that attribute-wise search indicates non-compensatory decision rules (see, e.g., Payne et al., 1993), although this assumption has been criticized for methodological reasons (Bröder, 2000). Furthermore, it has been argued that different psychological principles may be at work in both phases of the decision process (Betsch & Glöckner, 2010; Glöckner & Betsch, 2008a; Orquin & Müller Loose, 2013). Finally, decision theories rather tend to focus on one of the processes (search or integration) with the adaptive decision-maker model (Payne et al., 1988), the heuristics toolbox (Gigerenzer, Hertwig, & Pachur, 2011), and evidence-accumulation models (e.g., Busemeyer & Townsend, 1993) as notable exceptions. These approaches explain information acquisition and its integration simultaneously and on equal footing.

Glöckner and Betsch (2008a) proposed a spreading activation network model that formalizes judgment and decision making as a coherence-maximizing process. This parallel constraint satisfaction (PCS) model accounts for various empirical decision phenomena simultaneously, such as confidence ratings, choices, response times (e.g., Glöckner et al., 2014), and shifts in the perceived validity of predictive cues, which is a unique prediction of this model (Glöckner et al., 2010). However, PCS has also been criticized for focusing solely on information integration (Marewski, 2010), and in addition, it has been shown to fail in situations that require active information search (Söllner et al., 2013). As a consequence, Jekel et al. (2018) recently proposed an extension of the PCS model termed iCodes (integrated coherence-based decisions and search) that applies the same parallel constraint satisfaction mechanism of coherence-maximization to both information integration *and* search processes.

One major prediction of this model – the attraction search effect – has been confirmed in several studies (see details below). In the current work, we test an additional, more subtle prediction of iCodes, which states that activations of cue values due to exogenous influences will have both a direct (bottom-up) and an indirect effect (top-down) on search behavior. The original iCodes model explains the dynamic development of preferences or inferences based on the information structure and the cue validities through an automatic flow of activation through the network. Hence, information structure and cue validities are *endogenous* factors determining activation and therefore attention allocation. However, there will certainly also be *exogenous* factors that drive the allocation of attention and thus affect the activation pattern across the components of the decision representation. As one potential candidate of such exogenous influences on the activation of information, we manipulated the *visual salience* of cue values in the current experiments.

The article is structured as follows: First, we briefly describe the main idea of iCodes as well as empirical evidence relevant to it. In this section, we also explain the rationale for the predicted direct bottom-up and indirect top-down effects. Second, we give a brief overview of some attention/salience effects that have been reported in the decision literature. Third, we report a simulation study to substantiate the predictions, followed by three experiments testing top-down and bottom-up effects of salience on information search. Although the results were generally supportive of iCodes, the predicted indirect top-down effect of salience on search could not be detected. Finally, we conclude by discussing the theoretical implications of the findings.

## A coherence model of decision and search processes: iCodes

The assumption that people strive for a *coherent* representation of their physical and social environment is one of the most influential theoretical ideas in psychology, beginning with Gestalt principles of perceptual organization (Wertheimer, 1922/2017) and problem solving (Duncker, 1935) reaching out to fundamental views of balance and resolving cognitive dissonance in social psychology (Festinger, 1957; Heider, 1958). Coherence principles can be formalized in spreading-activation networks that settle into a minimum energy level after some time, thus simultaneously satisfying multiple constraints imposed by the network structure (e.g., Rumelhart et al., 1986; Thagard, 1989). These parallel constraint satisfaction (PCS) models have also been proposed as psychological mechanisms to explain why people are often able to process extensive and potentially conflicting information quickly (Glöckner et al., 2014; Glöckner & Betsch, 2008b, 2012). This rapid information integration can be better explained by parallel processing of information as also mapped in the network’s representation of information than by sequential integration of information as typically modelled in decision heuristics. However, PCS (e.g., Glöckner & Betsch, 2008a) has been restricted to information integration, neglecting the search process, which on the other hand is an integral part of the sequential heuristics of toolbox approaches (Gigerenzer, Todd, and the ABC Research Group, 1999; Payne et al., 1993). Hence, PCS had some explanatory advantages for situations with simultaneously provided full information (see Glöckner et al., 2014), but toolbox models obviously had a wider scope of application by including search as an additional explanandum.

**Fig. 1:**
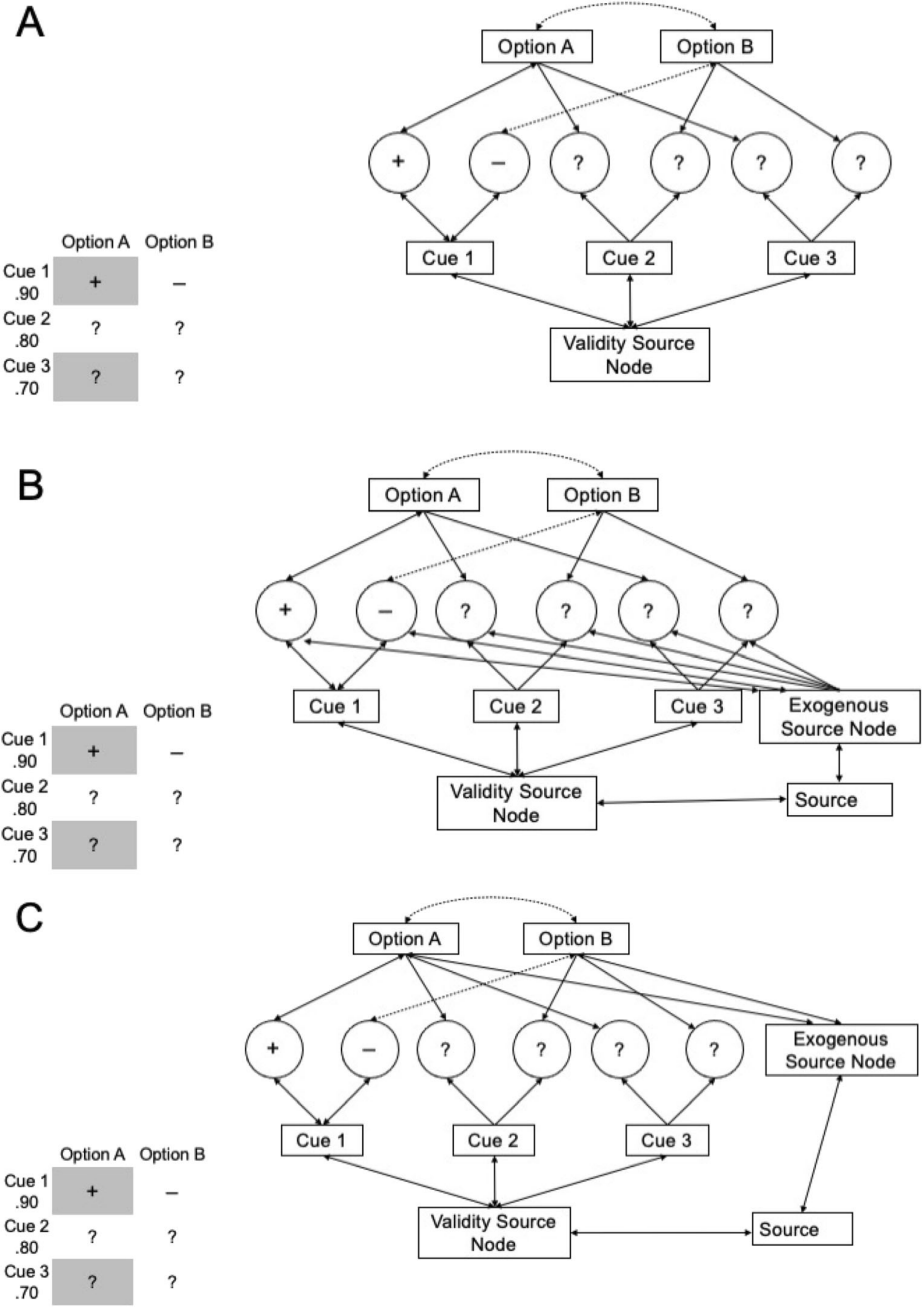
Modeling exogenous influences on information search within iCodes. Links in the network can be excitatory (solid lines) or inhibitory (dashed lines) and bi-directional (two arrow heads) or uni-directional (one arrow head). **a** Original network without exogenous influences. **b** Exogenous influences modeled at the cue-value level. Here, salient cue values receive more activation directly. **c** Post hoc version of iCodes with exogenous influences modeled at the option level. In this version, the options that contain any salient information receive more activation

Recently, Jekel et al. (2018) proposed iCodes, an extended PCS model that also includes search processes. A schematized version of iCodes is depicted in Fig. 1a, for a situation with three binary cues and two options. Cues, cue values, and options are represented as nodes, and activation is transmitted via connections between the nodes. Some of the cue values may already be revealed (see the “+” for Cue 1 for Option A), others may still be concealed (“?”). The strength of a connection is symbolized by line thickness, dashed lines represent inhibitory connections. The so-called “validity source node” has no psychological meaning, but it initiates the stimulation of the decision and search process in the model by pumping bottom-up activation into the network. First, this activation flows towards the cue nodes and the amount of activation they receive is proportional to their (perceived) validities (i.e., predictive value). In a next step, cue-value nodes receive activation and the more bottom-up activation an already opened cue value receives, the more activation (or inhibition, if the cue value is negative) it transmits “upwards” to the corresponding option. Hence, cue values belonging to more valid cues transmit higher amounts of activation to the option. This continued spread of activation to the options, however, is only initiated for already opened cue values – concealed cue values do not propagate activation further as they are only connected to options and cues via unidirectional links. Since the links between available cue values and options are bidirectional, activated options will transmit back some activation to their attached cues via their respective cue values, hence changing their relative activations. Concealed cue values are also connected to their respective option nodes. These connections are unidirectional in that they only “pass down” activation from the options and do not continue the spread of activation “upwards” to the options. As soon as a cue value becomes available by actively revealing it, the connection to the option becomes bidirectional, and the valence of the revealed value determines whether the connection is excitatory or inhibitory.^1^ The simple assumption of iCodes is that the concealed cue value with the highest activation will be most likely examined next. Activation of cue-values – and thus their predicted probability to be searched next – therefore depends on both bottom-up validity-driven activation as well as top-down option-driven activation.

One major prediction following from this architecture has been termed the *attraction search effect* (ASE; Jekel et al., 2018). A positively activated option will propagate back activation downwards to its associated cue values, and thus, they receive activation from options in addition to the activation from cues. An indirect effect of information search also follows from the model: The more activated option inhibits the less activated one, which, in turn, inhibits its respective associated cue values. Both influences thus increase activations of nodes representing concealed cue-values if the cue-values carry information on the positively activated option and decrease activations of cue-values linked to the negatively activated option. As iCodes predicts that the cue value with the resulting highest activation will be (most likely) searched next, this process will lead to the predicted tendency to continue information search for the currently most attractive option given the already revealed information. This ASE prediction has been confirmed in three experiments (Jekel et al., 2018) by using a similar stock market paradigm to the one used here (see below). Furthermore, the authors demonstrated this effect in five re-analyzed experiments using an information board paradigm. Finally, in three further experiments, Scharf et al. (2019) could show that the effect depends neither on the specific content domain (stock market) nor on the presentation format (matrix-like information board) that were used in the initial experiments. Hence, the ASE seems to be robust and fairly generalizable.

A second prediction, which we test here, is that *exogenous* activation of cue values should also have both a direct as well as an indirect effect on the activation of the other cue values and, thus, ultimately information search. In Fig. 1b, one possibility to implement these exogenous influence factors is presented. The implementation is based on the assumption that each piece of information could directly be activated by exogenous factors (i.e., information-based implementation of exogenous factors in iCodes; for alternative implementations see *Discussion*). Exogenous influences are represented by additional bottom-up links from an “exogenous source node.” Any concealed cue value “?” should have a higher probability of being opened if it receives additional activation from this exogenous source. This is what we will call a *direct* or *bottom-up* effect. Since we will use an index of the ASE as the empirical outcome variable in our experiments, this translates into the interaction hypothesis that the ASE should be stronger, if a “?” with additional exogenous activation is part of the more attractive option, whereas the ASE would be weaker if this “?” belongs to the less attractive option. However, like the prediction of the ASE itself, there should also be an *indirect* or *top-down* influence if the already revealed cue values (“+” or “– “) receive additional exogenous activation. Here, the ASE should increase if the salient cue value matches the overall attractiveness of the option (positive value for attractive option or negative value for unattractive option), whereas the ASE should decrease in mismatch cases (positive/unattractive or negative/attractive).

Of course, the notion of an exogenous influence is quite abstract, and one might wonder which psychological processes qualify as plausible candidates. Jekel et al. (2018) argued that *visual salience* of cues might be one such driver of cue activation since it is known that salience guides attention toward a stimulus, and it is the prototype of a bottom-up influence (see, e.g., Calvo & Nummenmaa, 2008; Pashler et al., 2001; Wolfe & Horowitz, 2017).^2^ Hence, in the experiments, we manipulated the relative salience of revealed and concealed cue values in order to test the above-mentioned hypotheses about direct and indirect effects of cue activation on search.

## Salience and attention effects in decision making

Following Jarvenpaa’s (1990) classic demonstration of presentation effects on decision making using graphical displays, the influence of visually salient attributes on decisions has repeatedly been demonstrated. For example, Sun et al. (2010) evoked preference reversals in a temporal discounting task depending on the relative visual salience of either the time dimension or the reward dimension in graphical displays. Bryant (2014) and Bröder et al. (2021) showed that salient cues are weighted more strongly in multi-attribute decisions. Kunar et al. (2017) showed that few salient items in a rapid serial visual presentation influenced final value judgments of the whole series. Milosavljevic et al. (2012) used a food preference task and found salience effects under restricted conditions involving rapid choices under cognitive load when only weak a priori preferences were present. One prominent theory that could account for effects of saliency at least in a post hoc fashion is the attentional drift diffusion model (aDDM; Krajbich et al., 2010; Krajbich & Rangel, 2011; Krajbich et al., 2012). The model assumes that the drift-rate of evidence accumulation is dependent on which option is fixated. Specifically, during the fixations to an option the value of this option is taken into account whereas the value of the other option(s) is considered at a discounted rate only. Hence, any manipulation of factors that increases the number of fixations to an option (such as saliency) should influence choice according to aDDM. In line with the model predictions, eye-tracking studies revealed that eventually chosen objects were fixated longer on average than non-chosen objects, and that the last fixated option had a higher probability to be chosen (Krajbich et al., 2010). However, the explicit computational incorporation of stimulus saliency into the model was accomplished by Towal et al. (2013), who predicted gaze patterns quite accurately with a model combining salience (bottom-up) and value influences (top-down) on gaze patterns in economic decisions.

The correlational evidence was backed up by experimental evidence in which the presentation times of objects were manipulated (300 vs. 900 ms) and longer times resulted in preference shifts towards the preferred object or away from the non-preferred object, thus demonstrating the *causal* role of attention allocation for preference strength (Armel et al., 2008; see also Ghaffari & Fiedler, 2018; Newell & Le Pelley, 2018; Pärnamets et al., 2015).

Probably the most established attention effect in choice is the *gaze cascade effect*: the option eventually chosen in preference tasks is increasingly fixated in the decision process (e.g., Shimojo et al., 2003). However, recently Sepulveda et al. (2020) showed that a simple task-framing change (using a choose-to-reject procedure) reversed both the cascade effect and the looking preference: The eventually chosen low-value option was fixated more and increasingly so towards the end of the trial. Hence, there is no simple correspondence between attention and option *value* but rather between attention and task-relevant information.

There are interesting conceptual similarities between the ASE predicted by iCodes and the gaze cascade effect in that both rely on the fact that the increasingly attractive option receives more attention. One fundamental difference, however, concerns the fact that ASE – as investigated so far – concerns mainly deliberate actions of active information search (e.g., using the mouse to open an information box). Gaze cascade effects, in contrast, are observed as trends in fixations that are arguably less voluntary. Recent work shows that the overall valuation of the options already exerts an influence on attentional direction quite early in a decision trial (Gluth et al., 2020), lending support to the idea of a positive feedback loop between attention and evaluation that is compatible with the basic assumptions of iCodes. In the current work, we investigate the ASE in a paradigm of active information search. We leave more in depth theoretical and empirical investigations of the relation of gaze-cascade effects and the ASE to future research.

Under the well-grounded assumption that visual salience drives attention, we test predictions that visual salience of cues will influence pre-decisional search as derived from an extended iCodes model with an exogenous influence factor.

## Simulating the predictions of the extended iCodes model

The experiments to be reported next aimed at testing the *direct* (bottom up) and the *indirect* (top-down) salience effects generated from the models. We substantiate these qualitative predictions with a simulation. We realized experimental conditions (detailed in the next section) that aimed to increase the ASE in comparison to a neutral, non-salient baseline condition by a direct salience effect (a salient hidden information marked “?” on the attractive option), or to decrease the ASE by highlighting a “?” on the less attractive option. Furthermore, there were four conditions testing indirect “top-down” effects by making positive (“+”) or negative (“−”) information salient, either on the attractive or the less attractive option. A match (salient “+/attractive” or “−/unattractive”) should increase the ASE as compared to baseline, whereas a mismatch (salient “+/unattractive” or “−/attractive”) should decrease its size according to the intuition derived from the model in Fig. 1b.

For the simulation, we included all eight cue patterns used in the experiments (see next section) and predicted the size of the ASE by running the extended iCodes model. There are two individual parameters in the model, namely *P*, which models the transformation of cue validities to subjective cue weights (controlling the extent of compensatory vs. non-compenstory cue weighting) and λ_s_, which controls the steepness of the sigmoid choice function transforming the activation difference between hidden cue values into a search probability. For both parameters, we used the mean values estimated from the samples in Jekel et al.’s (2018) original work for the simulation (*P* = 1.66, λ_s_ = 20.18). Hence, we used empirically obtained values from another study for the simulation. The decay parameter was increased from .1 to .2 in order to account for the effect of an additional node in the network and thereby avoiding ceiling effects for node activations. The top-down connections from options to hidden cue values were increased from .01 to .10 in order to emphasize potential top-down effects. The parameterization as well as simulation syntax is available at https://osf.io/8hkbf/.

**Fig. 2:**
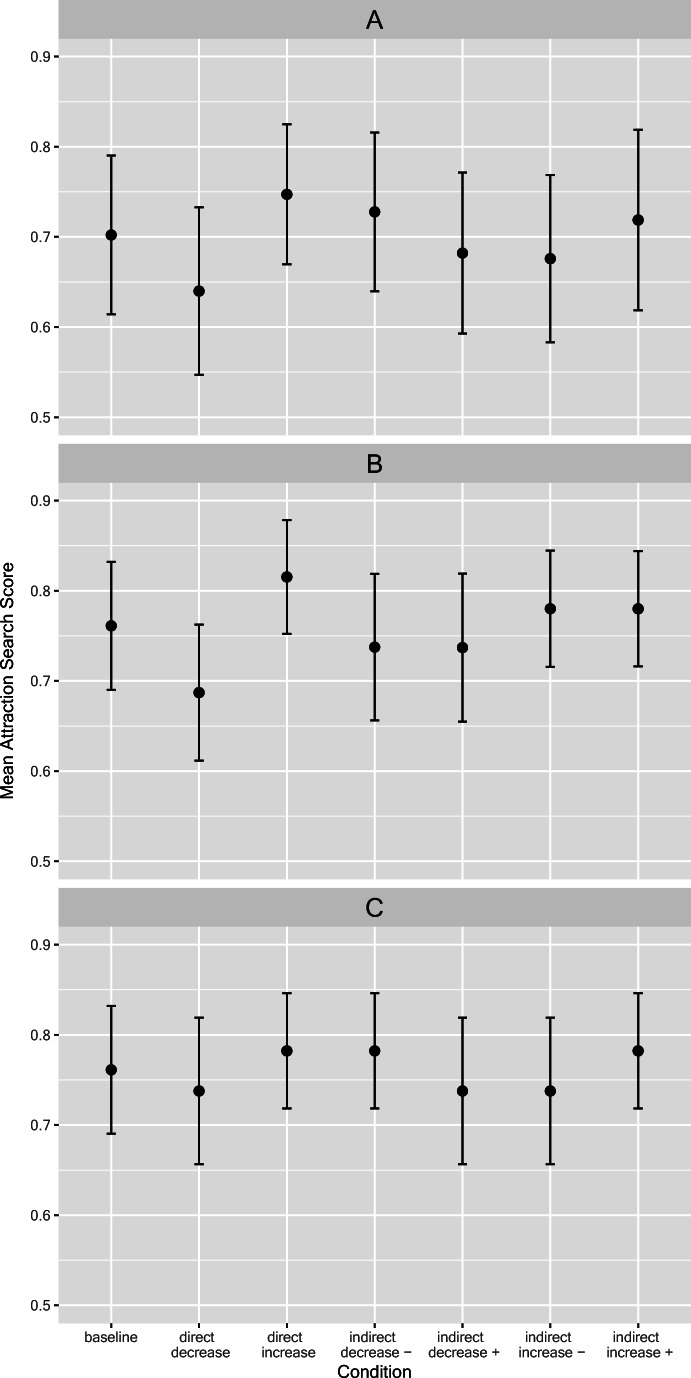
Mean size of the attraction search score (ASS) as observed in the experiments for each experimental condition (**a**), predicted size of the ASS by using the a priori extended iCodes model (**b**), and predicted size of the ASS by the post hoc model (**c**). Error bars are standard deviations across the eight stimulus patterns

The middle panel B of Fig. 2 presents the predicted size of the ASS for each of the seven experimental salience conditions, including the baseline condition. Note that the absolute size of the ASE is not of much interest here (i.e., the model parameter λ_s,_ moderating the absolute size of search probabilities was not fitted to the data), but rather the deviations of the salience conditions from the baseline without any salient cue. The simulation confirms the conceptually derived prediction that salience should have both direct and indirect effects on search behavior, resulting in increases or decreases of the ASE. Note also that according to the current parameterization, direct effects should be stronger than indirect effects.

## Experiments

The three experiments aimed at testing the prediction of a direct as well as an indirect or top-down salience effect in information search. In the studies, partially open information boards were used in which some (additional) information was concealed and could be opened by the participant before making a choice. When Experiment 1 failed to find an indirect salience effect, Experiment 2 elicited an additional, pre-search attractiveness rating from participants. By guiding their attention to a holistic evaluation of the options, this manipulation was intended to increase the amount of coherence-based processing. Experiment 3 used various degrees of visual contrast to manipulate salience within the experiment. All experiments were otherwise very similar, so we describe their methodology and results together and point to differences where appropriate.^3^

### Design and materials

A hypothetical stock-market game was used as a decision environment (e.g., Bröder, 2003; Newell & Shanks, 2003) in which two hypothetical stocks in each trial (with random three-letter names) were described by four binary cues denoted as brokers who could either recommend (denoted by “+”) or not recommend (denoted “−”) a stock. Brokers had different cue validities (sorted from top to bottom), and information was in part freely accessible, while two pieces of cue information were hidden (denoted “?”), one of which could be acquired during information search. Eight cue patterns were used as depicted in Table 1. In the table, all patterns are coded in Version 1 in which Option A (left column) is the more attractive option, given the revealed cue values. In a second version of each pattern, column entries were switched left versus right, so Option B was more attractive.

**Table 1:** Eight basic patterns used in the information board paradigm of all experiments. In these versions, option A is always the more attractive option

Pattern 1		Pattern 2		Pattern 3		Pattern 4	
Option A	Option B	Option A	Option B	Option A	Option B	Option A	Option B
?	?	+	-	+	+	-	-
+	+	-	-	?	?	+	-
-	-	+	+	+	-	?	?
+	-	?	?	-	-	+	+
Pattern 5		Pattern 6		Pattern 7		Pattern 8	
Option A	Option B	Option A	Option B	Option A	Option B	Option A	Option B
+	-	+	-	?	?	-	+
-	+	?	?	+	-	+	-
?	?	+	+	-	+	+	-
+	-	-	+	+	+	?	?

Salience was manipulated within subjects in an unbalanced design with one baseline condition in which no cue information was salient. In two direct *bottom-up* conditions, a concealed cue value (“?”) was presented in a visually salient manner either on the more attractive option or on the less attractive option (factor *option*). In four indirect *top-down* conditions, positive or negative values of cues were salient, on either the attractive or the unattractive option. This results in seven conditions altogether, exemplified with the first basic pattern of Table 1 in Table 2. Only one of the cue values was emphasized by salience in each decision trial. Two versions of the eight patterns in seven conditions were presented twice each, resulting in 2 × 8 × 7 × 2 = 224 decision trials for each participant. In Experiment 3, an additional between-subjects factor varied the strength of the salience difference by using different font colors on a white background (RGB value rgb(255,255,255) = HTML color name “White”) with a weak (rgb(169,169,169) = “DarkGray”) versus medium (rgb(128,128,128) = “Gray”) versus high contrast (rgb(0,0,0) = “Black”) between highlighted and regular cues (rgb(192,192,192) = “Silver”).^4^

**Table 2:**
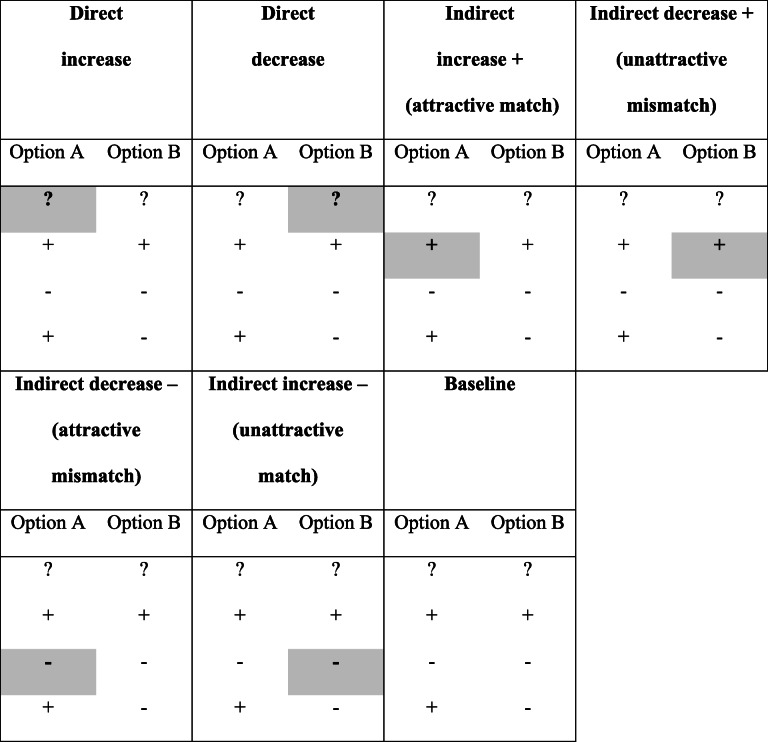
Seven Salience conditions for Pattern 1 of Table 1 with salient cue values marked by shading

In each trial, participants could open one of two concealed cue values with the same validity. Hence, validity influences on search were held constant within each trial. Of primary interest was whether participants acquired information about the attractive or the less attractive option, potentially indicative of an attraction search effect.

### Participants

To achieve a decent power, we aimed at 80 participants per experiment based on a priori power analyses (see pre-registration reports). For each of Experiments 1 and 2, *n* = 80 participants were recruited from the University of Mannheim, most of them students of different majors. Experiment 3 had *n* = 90 participants from the same pool randomly assigned to the three salience conditions (*n*_*low*_ = 31, *n*_*medium*_ = 30, *n*_*high*_ = 29). The mean age of the whole sample was 22.99 years (*SD* = 3.69, *range* = 18–47), 167 participants were female, 82 were male, and one did not provide gender information.

### Procedure

**Fig. 3:**
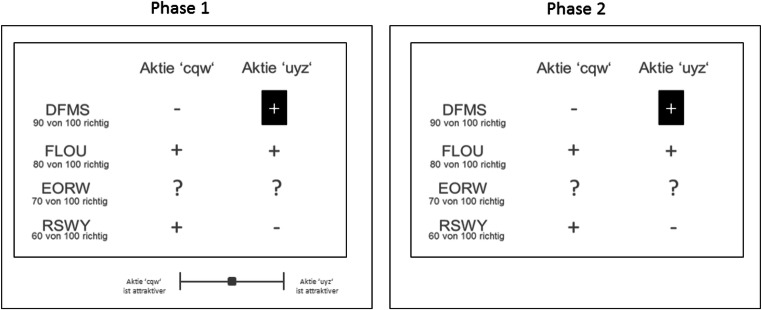
Screenshots of trials in Experiment 2. In Phase 1, participants judged the relative attractiveness of both options by using the ruler. After the judgment, they could acquire additional cue information in Phase 2 (i.e., request information hidden behind one of the “?”)

After the participants had filled out a consent form,^5^ the instructions told participants that they would invest in one of two stocks in each trial for which brokers with different prediction success rates (i.e., cue validities) made recommendations. They could acquire further information hidden behind a “?”, and they could choose which of the two information pieces to acquire before making a choice. A typical task trial from Experiment 2 is depicted in Fig. 3. Participants’ task was to increase their profit by maximizing the number of correct choices. A correct choice was defined via a naïve Bayes rule based on all information assuming independence of cues (Lee & Cummins, 2004). Participants received a show-up fee of 3.50 € in each experiment (approximately US$4.00), and they earned 0.025 € for each correct investment (i.e., in line with the naïve Bayes rule). One procedural difference was introduced in Experiment 2: Prior to each information acquisition, participants rated the overall attractiveness of the options based on the given information by moving a ruler with the mouse either to the left (Option A) or to the right (Option B; see Fig. 3, left panel). After the rating, the ruler disappeared, and participants could acquire one piece of information by clicking one of the hidden cue values. After information acquisition, they chose one option by clicking on the option name. Before the next trial in Experiment 2 was started, participants indicated how confident they were that they chose the correct stock, again by moving a ruler with the mouse either to the left (“not confident at all”) or to the right (“very confident”). There were five practice trials before the 224 actual trials started. After completing these trials, participants were thanked and received their earnings. The participation in all studies took on average about 45 min.

## Results

For all experiments, we first checked whether there was an overall ASE, the hallmark effect predicted by iCodes. The attraction search score ASS was defined as the difference between the probability of searching information about the attractive option minus the probability of searching the other option. If search and attractiveness of options are unrelated, the expected value of the ASS is zero or chance level, an ASE as predicted by iCodes results in values larger than zero, whereas negative values would indicate results in opposition to iCodes. In a second step, as preregistered, we report 2 × 2 within-subjects ANOVAs involving the factors *salience effect* (direct vs. indirect) and predicted *effect direction* (increase vs. decrease).^6^ The latter factor is coded in a way that a main effect in the correct direction (increase > decrease) would confirm the salience effects predicted by iCodes. However, if this effect is qualified by an interaction with the factor *salience effect*, this might hint to effect size differences between direct and indirect predicted effects (basically between the patterns with salient “?” for the direct effect and the patterns with salient “+” or “–” for the indirect effects). Finally, as a follow-up analysis, we report 2 × 2 within-subjects ANOVAs with the factors *option* (salient cue on *attractive* vs. *unattractive* option) and *value* (salient cue value *positive* vs. *negative*) for the indirect cuing conditions only to check for potential asymmetries between positive and negative salient cue values.

### Overall attraction search effect

**Fig. 4:**
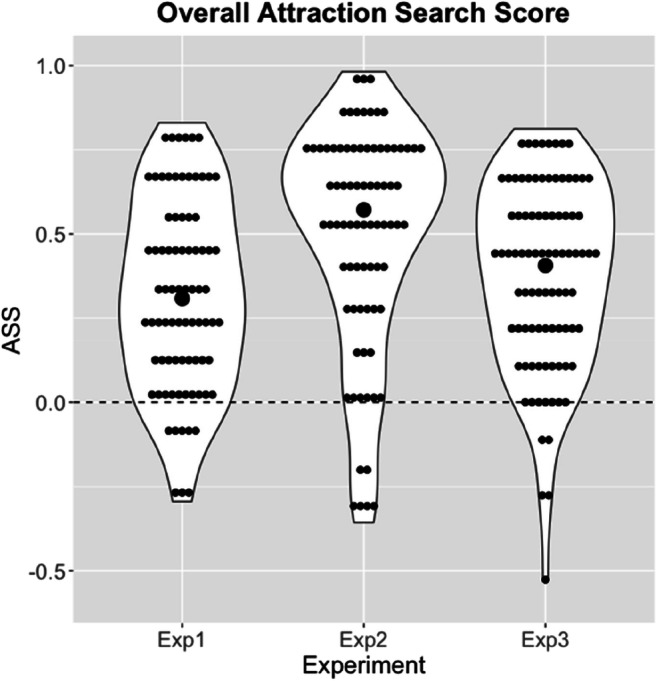
Violin plots showing the frequency distributions of the overall attraction search scores in the three experiments. Dashed line denotes independence of search and option attractiveness, higher values denote a stronger attraction search effect. Large dots denote median

Figure 4 shows the frequency distributions in violin plots for the overall ASS scores in the three experiments (*Ms* = .32, .498, and .38; *SD*s = .28, .33, and .28, respectively). All scores are significantly greater than zero (*t*(79) = 10.14, *t*(79) = 13.55, and *t*(89) = 12.81, all *p*s < .001, all Cohen’s *d*s > 1.13). The picture is virtually identical if we restrict this analysis to the baseline condition without any salient cues (all *t*s > 8.85, all *p*s < .001, all *d*s > 0.99). Hence, the hallmark prediction of iCodes was again confirmed in all three experiments: People will preferentially search information about the currently more attractive option.

### Salience effects

**Fig. 5:**
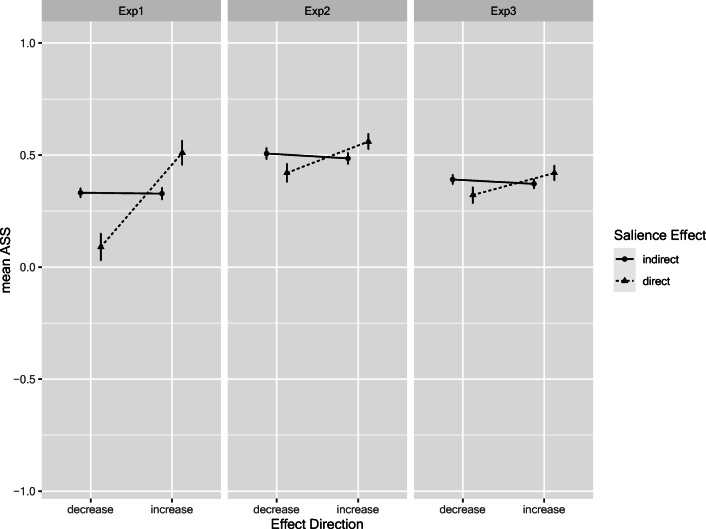
Attraction search scores as a function of predicted effect direction, and type of salience effect (direct vs. indirect) predicted by iCodes in the three experiments. Error bars denote 95% confidence interval based on within-subjects error

We conducted 2 × 2 within-subjects ANOVAs on the ASS involving the factors *salience effect* and *effect direction*.^7^ For Experiment 3, we added the between-subjects factor *salience strength* (weak, medium, strong). However, since this manipulation had no main or interaction effect at all (all *F*s < 1.9, all *p*s > .16, all η_p_^2^ < .045), we collapsed data across this factor for ease of presentation. Figure 5 shows the mean ASS values for these analyses. There was a small but significant (direct vs. indirect) *salience* main effect only in Experiment 1, *F*(1,79) = 6.31, p = .014, η_p_^2^ = .07, but not in the other experiments, *F*s < 1, both η_p_^2^ < .01. Note, however, that the somewhat larger ASS values for the direct salience effect in Experiment 1 do not have a specific interpretation. However, in all three experiments, there was a predicted main effect of *effect direction*, *F*(1,79) = 53.14, *F*(1,79) = 9.39, and *F*(1,89) = 5.95, respectively, all *p*s < .02. (η_p_^2^ = .40, η_p_^2^ = .11, and η_p_^2^ = .06, respectively). Hence, the ASS was larger when the inclusion of the salient cue according to iCodes should lead to an increase in ASS, whereas it was smaller if a decrease was predicted. However, significant interactions in all three experiments qualified this result, all *F*s > 12.5, all *p*s < .001, all η_p_^2^ > .12. Follow-up *t*-tests showed that the predicted ASS differences only emerged for the direct bottom-up effects (hence, the salient “?”), *t*(79) = 8.57, *t*(79) = 4.497, and *t*(89) = 3.41, as well as *d*s = 0.96, 0.50, and 0.36, respectively, all *p*s < .001, but not for the hypothesized indirect top-down influence (hence, the salient “+” or “–”). The corresponding *t*-values for the top-down effects were *t*(79) = 0.22, *t*(79) = 1.13, and *t*(89) = 1.24, all *p*s > .20, all *d*s < 0.14 . Since the lack of an indirect effect is potentially consequential, we pooled all three experiments yielding a power of .95 to detect a “small” effect *d*_z_ = 0.2 according to Cohen’s (1988) conventions. However, this resulted in *t*(249) = 1.51 and *p* = .13, *d* = .096, still pointing to the absence of even a small effect.^8^

### Testing for asymmetries between positive and negative values

**Fig. 6:**
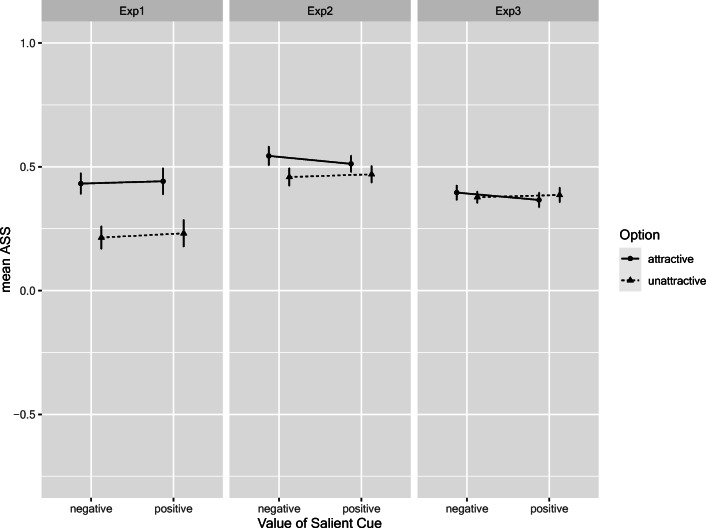
Attraction search scores as a function of value of salient cue and option attractiveness. Error bars denote 95% confidence interval based on within-subjects error

For the subset of conditions with salient positive or negative cue values, we tested whether they would exert a different influence on search. Hence, we coded whether the salient cue was on the attractive versus unattractive option (factor *option*) and whether the cue value was positive or negative (factor *value*), and analyzed the ASS with a corresponding 2 × 2 within-subjects ANOVA. Results are depicted in Fig. 6. There was a significant *option* main effect in Experiments 1 and 2, *F*(1,79) = 35.74 (η_p_^2^ = .31) and *F*(1,79) = 9.62 (η_p_^2^ = .11), both p < .003. However, such an effect was missing in Experiment 3, *F*(1,79) = 0.002 (η_p_^2^ < .001). More importantly, there was neither a main effect of *value* nor any interaction of the factors, all *F* < 1.55, all *p*s > .20, all η_p_^2^ < .02. The latter results show that there was no asymmetric influence of negative and positive values when they were salient. However, in Experiments 1 and 2, the salience per se (independent of the cue valence) triggered search in a bottom-up fashion: A salient cue on the attractive option would lead to more search on that option (thus boosting the ASS), whereas a salient cue on the unattractive option would lead to less search on the other option (thus reducing ASS). Note, however, that the ASS is still larger than zero, demonstrating a strong ASE. Making the unattractive option more salient only reduced this preference in favor of the attractive option by a small amount.

### Attractiveness ratings and confidence ratings in Experiment 2

In Experiment 2, attractiveness ratings for the options were elicited to enhance coherence-based processing. These allow for testing our attractiveness manipulation: If the scale is coded from −100 (perfect preference for less attractive option) to +100 (preference for attractive option) with 0 as the indifference point, we obtained a mean rating of 39.25, which is well above zero, *t*(79) = 18.96, *p* < .001, *d* = 2.12. Furthermore, the means ranged from 5.08 for Pattern 8 (see Table 1) to 54.8 for Pattern 2. According to naïve Bayes, the odds in favor of the more attractive options for Patterns 8 and 2 are 1.04 and 9.00, respectively. Indeed, the mean attractiveness ratings closely correspond to the Bayesian odds with Spearman’s ρ = .976 across the eight patterns. Hence, participants readily identified the more attractive options in each pattern, and they were also sensitive to the attractiveness differences across patterns. Similarly, the mean confidence ratings elicited in Experiment 2 after each choice followed the odds of the patterns closely, Spearman’s ρ = .88, and they were higher if participants chose in line with naïve Bayes (*M* = 64.23) as compared to choosing incorrectly (*M* = 45.10), *F*(1,79) = 196.09, *p* < .001, η_p_^2^ = .71, with an overall mean value of 60.95. Furthermore, for each of the eight stimulus patterns, the extended a priori iCodes model predicts a unique difference of activations between the options for each of the eight patterns and seven salience conditions. These predictions correlate with the (8 patterns × 7 conditions =) 56 confidence means very well with *r* = .798 (see the Appendix for details).

## General discussion

The main research questions tackled in this paper were: (1) whether the attraction search effect predicted by iCodes can be replicated, (2) whether there is a bottom-up (direct) influence on cue search through making some cue values salient, and (3) whether there is an additional top-down (indirect) influence of increased or decreased option coherence triggered by salient cue values. Three experiments with partially opened information boards were conducted in which hidden (“?”), positive (“+”) or negative (“–”) cue values were made visually salient either describing the more attractive or the less attractive options. Experiments 2 and 3 were basically replications of Experiment 1 that tried to increase coherence effects by requiring an overall attractiveness rating for the options before information search (Experiment 2) or to enhance visual salience (Experiment 3). The main results of the experiments were fairly consistent and can be summarized as follows: There was a clear and strong attraction search effect, one major prediction of the iCodes model. Second, there was a clear demonstration of a bottom-up salience effect in the sense that options that contained a visually salient cue value were searched more than options without a visually salient cue value. This is reflected in the main effect of salience on the ASS in the ANOVAs for all cue valences (?, +, –) in Experiments 1 and 2 and the main effect for “?” in Experiment 3. This effect is compatible with iCodes, but it is admittedly not a prediction unique to this model. Any theory that allows for attention-grabbing aspects of visual salience might predict such an effect. This result is only problematic for toolbox approaches assuming the application of strictly validity-based heuristics. Finally, the results were quite consistent in suggesting the absence of a top-down effect that we would expect for valenced, salient cue values: Since they either strengthen or weaken the coherence of an options’ representation, we would have expected a moderation of the strength of the ASE. Combining the data from all experiments yielded a power of 1-β = .95 to reject the alternative hypothesis of even a small effect in favor of the null hypothesis and an additional Bayesian analysis also showed evidence for the H0. Hence, although evidence for the main predictions of iCodes was found, the more subtle prediction was not supported by the results. Before discussing the theoretical implications of the findings, we briefly comment on some other aspects of the data.

### Other results

After we learned about the absence of a top-down coherence effect in Experiment 1, one goal of Experiment 2 was to enhance coherence-based processing by requiring participants to provide overall attractiveness ratings for the options prior to further information search. Although comparisons between experiments have to be taken with a grain of salt, a comparison of the overall ASS values between the experiments (M = .32 vs. M = .49, see Fig. 4) suggests that this measure was successful, *t*(158) = -3.64, p < .001, *d* = 0.58. We hypothesize that the attractiveness judgment focuses participants’ attention on a holistic evaluation of the options that in turn emphasizes coherence processing and hence influences search. However, before over-interpreting it, the effect remains to be replicated in a more controlled randomized experiment.

Experiment 3 was an attempt to explore the importance of salience by using a manipulation of its strength. However, contrary to expectation, this manipulation had no effect on behavior whatsoever. The interpretation must be post hoc, of course, but after the fact it appears plausible that in this paradigm, the effect of salience is probably rather qualitative than quantitative and thus non-gradual. Even in the weak contrast condition, the salient cues were highlighted and thus more visible in comparison to the others, thus probably grabbing some attention. The regular cues were printed in “Silver” on a white background. All salient conditions used colors that were clearly distinguishable from this color (dark gray, gray, and black). A higher contrast probably does not further increase the qualitative nature of this attentional influence, especially since the manipulation was between subjects, which obscured *differences* in salience.

As expected, we found a clear bottom-up salience effect in the conditions with highlighted concealed cue values (denoted by a “?”) in all three experiments. Somewhat unexpectedly, we also found a bottom-up main effect of the valenced cue values (“+” and “–”) without any interaction with option attractiveness in the first two experiments. Hence, having a salient cue increases an option’s probability of being searched, independent of the cue’s influence on option coherence. It is currently unclear, however, why this effect was absent in Experiment 3. Since Experiment 3 was procedurally almost identical to Experiment 1, we know of no ready explanation for this difference in results. Hence, the current evidence does not readily allow us to decide whether two results are false positives or one result is a false negative. The information-based implementation of exogenous influences in iCodes described so far would predict no effect, but an alternative option-based implementation described below would predict such an effect.

### Theoretical implications

The confirmation of a strong ASE in all experiments adds to the growing evidence that this effect is stable across various experiments as well as reanalyzed experiments (Jekel et al., 2018) and also across different content domains and display modes of the information (Scharf et al., 2019). As Jekel et al. have argued, the ASE is hard to reconcile (if at all) with heuristic models of search and decision making that typically rely on fixed search and stopping rules (Gigerenzer et al., 1999; Todd et al., 2012). Also, evidence accumulation models would have to be augmented by assumptions about the dependence of the search direction. For example, Gluth et al. (2020) have recently proposed such an extension of the attentional Drift Diffusion Model that accounts for the fact that gaze patterns were clearly influenced by a top-down influence of the options’ values: Options with higher subjective values were fixated longer and more often. This is similar to the attraction search effect that has been demonstrated with active information search up to now. Compared to this augmented model, however, the prediction of an ASE naturally follows from the architecture of iCodes.

As one flagship effect predicted by the model, it has been argued that the prediction of the ASE itself is not new, but resembles motivational as well as cognitive confirmation bias (also termed a positive testing strategy; Klayman & Ha, 1987), the phenomenon called pseudodiagnosticity (Doherty et al., 1979), or leader-focused search (DeKay, 2015). A motivational confirmation bias cannot explain our findings, however, since the paradigm does not allow the participant to foresee whether the next information confirms or disconfirms a favored hypothesis. With respect to a positive testing strategy, leader-focused search or pseudodiagnosticity, we agree that the ASE is very similar to these phenomena, but we think that the iCodes model provides a principled theoretical explanation based on coherence and a computational implementation, whereas “positive testing strategy” or “pseudodiagnosticity” arguably lack formalized explanations of the phenomena. Note, however, that DeKay (2015) offered a connectionist explanation for “leader-focused” search at the conceptual level without formal implementation, which is quite similar to the conceptual idea of iCodes.

An important theoretical question is why the predicted indirect salience effect did not appear. A potential answer could be that the implementation of the exogenous influences in the network model as depicted in Fig. 1b was incorrect. We initially assumed that salient cue values receive additional activation from an exogenous source node and that, therefore, their information receives more weight in the information-search process. This way of modeling exogenous influences on information search, however, is incompatible with our results: while the direct salience effect of highlighted, concealed information can be directly derived from this model set-up, the absence of the indirect salience effects of highlighted available information as well as their main effect in Experiments 1 and 2 cannot be reconciled with the suggested information-based implementation of the exogenous source node.

Thus, an alternative way of modeling exogenous influences on information search is warranted. One potential implementation that might be consistent with the pattern of results is to add the exogenous influence on the option level with connections from the exogenous source node to the option nodes directly, that is, an *option-based* implementation of exogenous influences. This idea is depicted in Panel C of Fig. 1. From this network structure follows that whenever a concealed or open cue-value is salient for one option, the option as a whole receives additional activation in the model and, due to the top-down effects on search, cue values with information on this option are more likely to be searched for. Such a model could probably explain the direct effects on search in our studies as well as the valence-independent effects in Experiments 1 and 2. It could also account for the fact that we did not observe an indirect effect. Since options are activated independently of whether a positive or negative cue value is highlighted, the assumed indirect effects that would be expected by our initial information-based exogenous influence should not be generated in the alternative option-based implementation. The model would imply that salience of information is rather processed on the level of options and not on the level of the information itself. In order to see whether this post hoc model assuming a direct salience influence on options would be compatible with our results, we implemented a simulation with this post hoc model. We thereby used the same model function and parameter values as for the a priori model with the weights for the additional links from the salience node to the option nodes set to .01. The predictions of such a model are depicted in Panel C of Fig. 2. As is obvious from visual inspection, this post hoc pattern of ASS across experimental conditions is much more similar to the actual data (depicted in Panel A of Fig. 2) than the original extension of a salience influence at cue level we used to generate the predictions for our experiments (Panel B). Still, the post hoc model is not perfect since it does not capture the descriptive size difference of effects in indirect and direct conditions that is apparent in the data.

It should be noted, however, that in all three experiments pieces of information belonging to one option were displayed in close proximity on the left or right side of a cue-matrix, respectively. Therefore, a second explanation for the observed results might be that salience guides attention towards one side of the cue-matrix. Future research could test these two opposing post-hoc hypotheses and the underlying option-based versus side-based implementation of exogenous influences. Both implementations could, for example, be tested by positioning cue-values of the same option at different places on the screen to disentangle effects of option activation and side activation.

A potential further explanation for the absence of the indirect salience effects is that the manipulation was too weak to trigger these effects. A theoretically more interesting explanation could be that our manipulation triggered a deliberate, top-down influence of salience on information search. The reasoning behind this idea is that due to the cue-value patterns we chose in these studies, participants could only search for one of two equally valid cue values. Thus, the only information participants had to guide their search was the attractiveness of options and the salience of information. This effect on search was intended, as the cue-value patterns were deliberately designed to keep validity influences constant and, thus, to increase the chances to observe an interaction of attractiveness and salience influences. Yet, in the light of it being observed that some participants show a tendency to search strategically (Jekel et al., 2018; Scharf et al., 2019), it is possible that participants perceived any salient cue-value as an implicit recommendation of the option and used this information to guide their search (cf. a more deliberate version of the option-based implementation of exogenous influences discussed above). Finally, in order to generate our initial predictions, we increased the weight parameter for the links between options and concealed cues. This was done to emphasize potential top-down effects. Hence, one further explanation for the missing indirect effects may be that they exist, but with other parameter settings they are too weak to be detected with standard sample sizes in experimental psychology. However, lacking relevant data, we currently prefer the post hoc model rather than accepting this potential self-immunization of the a priori model.

The current article demonstrates and tests how exogenous sources that influence attention can be included in iCodes to develop and test (more) comprehensive formal models for decision making and search (cf. Orquin & Müller Loose, 2013). Our results indicate that the initially assumed cue-level implementation of exogenous influence factors could only partially account for the findings. Model variants of iCodes assuming option-based or side-based exogenous influence instead of (or in addition to) information-based implementations could account better for the overall findings.

Various versions of the iCodes model including exogenous influence factors can be used for deriving quantitative predictions for a wealth of (exogenous) sources that have been shown to influence attention and choices. Potentially relevant factors include: (1) surface size of options (see Peschel & Orquin, 2013, for a review), position effects concerning (2) the center position (Atalay et al., 2012) and (3) the top and left position due to usual reading direction (Glaholt et al., 2010; see also Fiedler & Glöckner, 2012), and (4) manipulation of fixation durations for options (e.g., Armel et al., 2008; see also Ghaffari & Fiedler, 2018). For example, in the current experiments, the main effect of preferring to search the left option – probably due to the usual reading direction of our participants – was of comparable size as the direct salience effect (η^2^ = .058 and η^2^ = .047, respectively, when contrasting the two direct effect conditions). As this certainly incomplete list demonstrates, the common denominator of these exogenous factors is their ability to direct attention based on either simple perceptual factors (salience) as well as perceptual grouping or habits like reading. Other influences on attention allocation may be incorporated as well in future investigations.

The extension of iCodes by including bottom-up effects as exemplified in the current article seems to be a promising avenue for future research to develop and test more comprehensive formal models for decision making and search. Implementations of iCodes that assume option-based instead of or in addition to information-based exogenous influences are particularly supported by the data presented in this article.

One challenge for iCodes in general is the newly presented evidence by Sepulveda et al. (2020) demonstrating that attention allocation in gaze tasks can be reversed by switching from a choose-to-select task to a choose-to-reject task. Whether and how this effect can be reconciled with iCodes remains to be investigated. Also, iCodes’ ability to predict gaze behavior rather than active and costly search is yet untested. But it is clear that for tackling the effects by Sepulveda et al. (2020), iCodes has to be augmented with explicit assumptions about either transforming the interpretation of cue valence at the perceptual level or by changing the criterion for the next cue value to reveal in active search.

## Figures and Tables

**Fig. 7:**
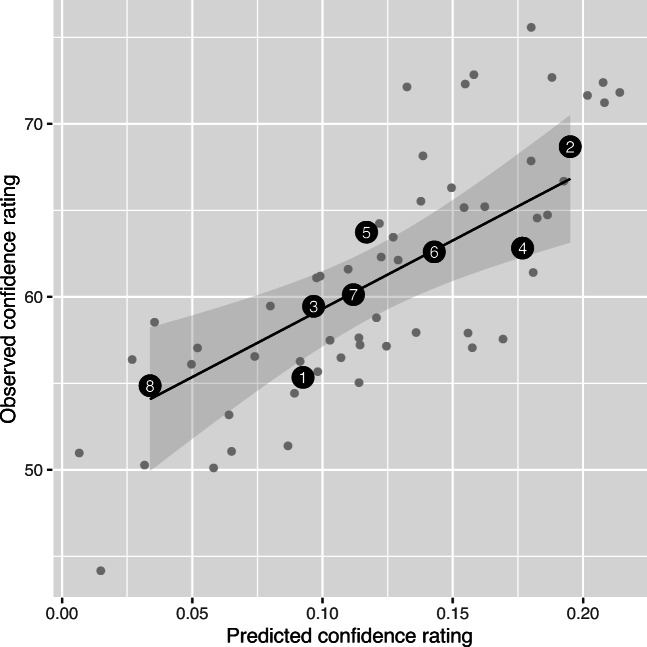
Predicted confidence ratings (i.e., differences in activations of option nodes) derived from the a priori extended iCodes model (x-axis) and mean observed confidence ratings (y-axis) for the eight stimulus patterns 1–8 (black dots) and 8 stimulus patterns × 7 salience conditions = 56 mean confidence ratings (grey dots). The regression line with indication of the 95% confidence interval is based on the confidence ratings of the eight stimulus patterns (i.e., black dots)

**Fig. 8:**
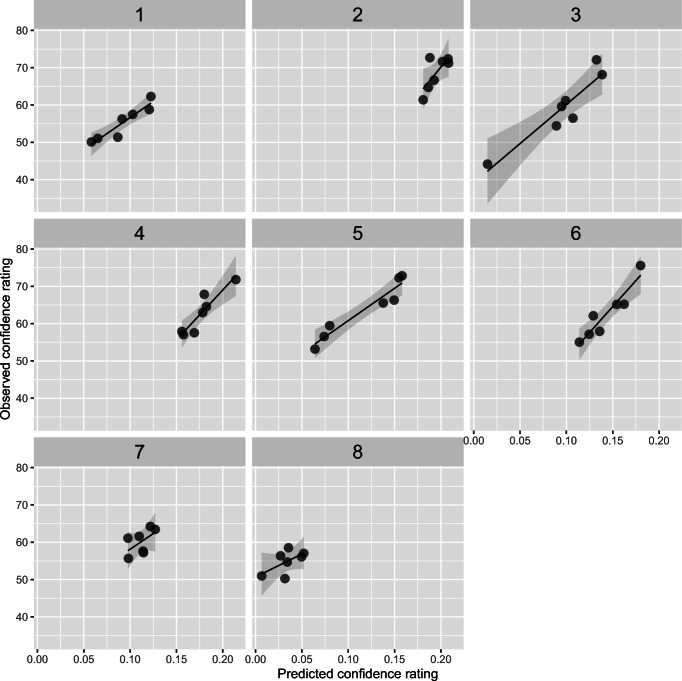
Predicted confidence ratings (i.e., differences in activations of option nodes) derived from the a priori extended iCodes model (x-axes) and mean observed confidence ratings (y-axes) for the eight stimulus patterns plotted in displays 1–8 and seven salience conditions (dots within pattern displays) plotted in each display. The regression lines with indication of the 95% confidence intervals are based on the confidence ratings of the seven salience conditions for each pattern.

## Appendix

According to PCS-DM and iCodes, the final difference of the node activations representing the options after the network has stabilized predicts decision confidence (Glöckner et al., 2014). As depicted in Appendix Fig. 7, confidence predictions of the a priori extended iCodes model indeed correlate with observed confidence ratings for the eight stimulus patterns (*r* = .883, *p* < .01) and for the 8 stimulus patterns × 7 salience conditions = 56 confidence ratings (*r* = .798, *p* < .001). As depicted in Appendix Fig. 8, regression lines with positive gradients for all stimulus patterns further indicate that prediction accuracy does not depend on specific stimulus patterns. This speaks for the generalizability of the model predictions beyond the specific stimulus patterns used in the current studies. The tight match between predictions and observations also shows when using individual and thus non-aggregated confidence ratings: The variable “confidence predictions” is a statistically significant predictor (*p* < .001) of the observed confidence ratings in a multilevel regression model including random intercepts for participants, stimulus patterns, and salience conditions. The predictor is also significant when the random intercepts of stimulus patterns and salience conditions are removed or are included as fixed effects instead. Thus, conclusions also do not depend on the aggregation level of the data and the specifics of the statistical analysis. Overall, results show that the extended iCodes model can predict participants’ confidence ratings already very well by using an out-of-the-box default parameterization.

## Footnotes

1 With this dynamic change of a link’s nature, the current model goes beyond the assumptions of common neural network models. Whereas a *physical* network realizing such a mechanism would probably require some complex gating mechanism to accomplish such a switch, the *conceptual* idea can be easily incorporated formally. Currently, we see iCodes as a formal conceptual theory for explaining coherence effects in decision making and search.

2 We do not want to touch the issue whether bottom-up influences like salience can be overridden by top-down control in specific instances. We rely on the agreed-upon notion that relatively salient stimuli are *ceteris paribus* typically more attended to than less salient stimuli.

3 Preregistration reports were uploaded at https://osf.io (Experiment 1: https://osf.io/2uspd/; Experiment 2: https://osf.io/u5hq7/; Experiment 3: https://osf.io/vr6np/ ). For convenience, all materials and data for the three experiments as well as copies of the pre-data reports are available at https://osf.io/8hkbf/. For brevity, we confined the analyses reported here to the ANOVAs mentioned in the preregistrations, skipping the mostly redundant regression analyses mentioned in the preregistration plans.

4 Unfortunately, the HTML color named “DarkGray” is actually lighter than “Gray,” which may confuse readers, but we chose to present the HTML names together with the RGB codes for appropriate referencing (e.g., see https://htmlcolorcodes.com/, retrieved on 2/9/2021).

5 Since participants were fully informed that they could interrupt the experiment any time without disadvantage and neither deception nor potentially harmful procedures were used, the study did not require approval by the IRB of the University of Mannheim according to the IRB’s regulations.

6 Coding of effect direction: An *increase* of the ASE is predicted for salient “?” on the attractive option, a *decrease* for a salient “?” on the unattractive option (direct effect). For the indirect effect, an *increase* of ASE is predicted for a *match* between salient cue value and option (“+” on attractive or “–” on unattractive option), whereas a *decrease* is predicted for a mismatch between cue value and option. “Decrease” and “increase” conditions are contrasted by the *effect direction* factor.

7 ANOVAS were conducted using the *afex* package for R (Singmann et al., 2020).

8 An additional undirected Bayesian paired-samples t-test (Jeffreys, 1961; Rouder et al., 2009) with a default Cauchy prior width r = 0.707 supports this conclusion: The data are 4.60 times more likely under the H0 than under the H1. In a robustness check, the Bayes factor increases for a wide Cauchy prior (r = 1) to 6.42 and for an ultrawide Cauchy prior (r =$\sqrt{2}$) to 9.03 for H0.
